# Physical Activity and Cancer Care—A Review

**DOI:** 10.3390/cancers14174154

**Published:** 2022-08-27

**Authors:** Weronika Misiąg, Anna Piszczyk, Anna Szymańska-Chabowska, Mariusz Chabowski

**Affiliations:** 1 Student Research Club No. 180, Faculty of Medicine, Wroclaw Medical University, 50-367 Wroclaw, Poland; 2 Department of Internal Medicine, Occupational Diseases, Hypertension and Clinical Oncology, Wroclaw Medical University, 50-556 Wroclaw, Poland; 3 Department of Nursing and Obstetrics, Faculty of Health Science, Wroclaw Medical University, 51-618 Wroclaw, Poland; 4 Department of Surgery, 4th Military Teaching Hospital, 50-981 Wroclaw, Poland

**Keywords:** cancer care, physical activity, survivors, quality of life

## Abstract

**Simple Summary:**

The aim of this paper is to outline the role and potential benefits of physical activity for cancer patients. We present a review of publications on the subject in order to compare the findings reported in the literature and draw general conclusions that could help clinicians who provide cancer care to develop a more comprehensive treatment approach. This review may also help patients overcome barriers and become more motivated to take up physical activity, which would improve their quality of life. We wish to demonstrate to patients that physical activity should not be regarded as a burdensome medical recommendation but rather as a factor that can reduce the risk of cancer mortality and recurrence.

**Abstract:**

In 2020, 19.3 million new cancer cases were diagnosed, and almost 10 million deaths from cancer were recorded. Cancer patients may experience fatigue, depression, anxiety, reduced quality of life and sleep problems. Cancer treatments cause numerous side effects and have a negative impact on all body systems. Physical activity is important for cancer patients. The aim of this review is to analyse recent studies on the role of physical activity in cancer patients and emphasize its importance. The review included 36 papers published in English between 2017 and 2021. The findings from these studies show that physical activity decreases the severity of side effects of cancer treatment, reduces fatigue, improves quality of life, has a positive impact on mental health and improves aerobic fitness in cancer patients. Moreover, it reduces the risk of cancer recurrence and death. Physical activity is recommended for patients with any type of cancer and at all stages of treatment. The type of physical activity should depend on the condition of the individual patient. It is extremely difficult to determine what type, intensity and duration of physical activity is likely to have the greatest effect.

## 1. Introduction

In 2020, 19.3 million new cancer cases were diagnosed, and almost 10 million deaths from cancer were recorded [1]. Cancer patients may experience fatigue, depression, anxiety, reduced quality of life (QoL) and sleep problems [2,3,4].

Cancer treatments have many side effects. They exert a negative impact on: the cardiovascular system, the endocrine system, the digestive system, the immune system, the nervous system, the respiratory system, systemic symptoms such as fatigue, which can persist for many years after treatment, and lymphedema [5].

Physical activity (PA) is important for cancer patients. The World Health Organization distinguishes between two types of physical activity: aerobic physical activity and anaerobic physical activity. Physical activity can be classified according to intensity as: light-intensity physical activity, 1.5–3 metabolic equivalents of task (METs), which does not result in a significant increase in heart rate or respiratory rate (one example of light-intensity physical activity is slow walking); moderate-intensity physical activity, 3–6 METs; and vigorous-intensity physical activity, more than 6 METs [6].

PA improves QoL, increases aerobic fitness, has a positive influence on mental health and reduces the side effects of cancer treatment, fatigue and mortality in cancer patients [2,3,7,8,9]. The type of physical activity should depend on the condition of the individual patient. A patient’s response to a given physical activity stimulus may vary due to the side effects of treatment, demographic factors (age), mobility restrictions or comorbidities [10]. However, patients should undertake physical activity unless the disturbances are severe enough to prevent them from exercising [11]. Moreover, a patient’s ability to tolerate exercise may vary during a disease. This is caused by the variability in the intensity of the symptoms [5].

With patients facing a life-threatening illness, recommending additional physical activity may seem to be unnecessarily burdensome or too simplistic, as it would require an investment of time and energy from the patient [10].

Although there are many research papers about the benefits of physical activity in cancer patients, in practice combining PA with treatment is rare. In 2020, as many as 35.5% of the cancer survivors aged 18 years and older reported physical inactivity [12]. Only 7% of cancer patients perform adequate exercises [13]. This manuscript analyses the recent studies from the last five years on the role of physical activity in cancer patients under active treatment and cancer survivors and emphasize its importance. The review summarizes the results gathered from 36 articles and presents the influence of PA in cancer care for different types of tumour and patient groups.

The aim of the study is to present the impact of physical activity on cancer patients and cancer survivors in order to reach the largest possible group of readers, both among healthcare professionals and oncological patients.

## 2. Material and Methods

**Figure 1:**
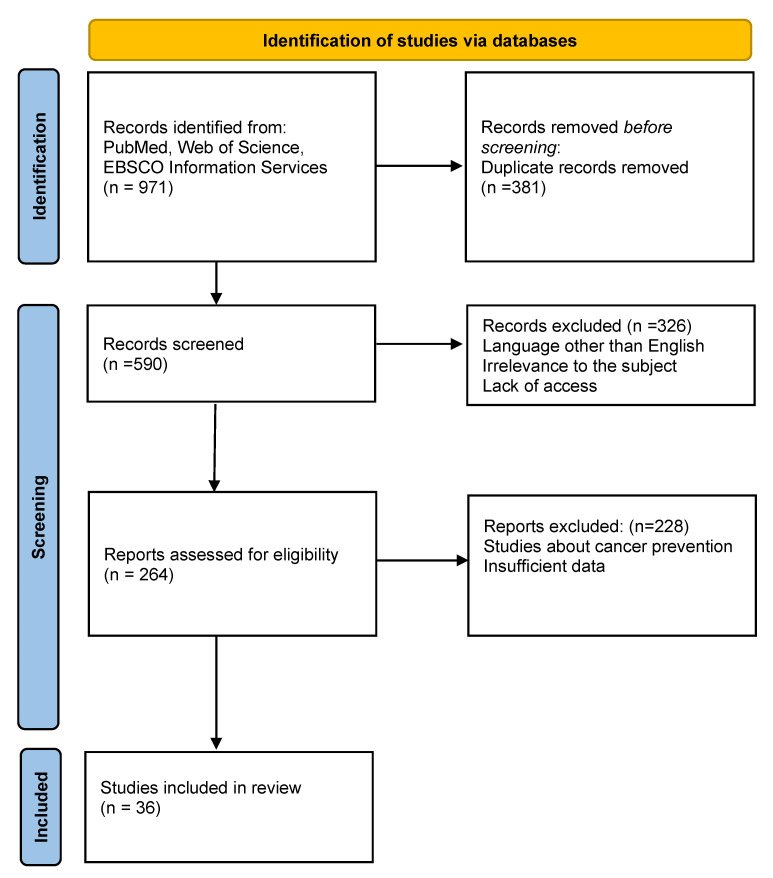
Identification of studies via databases.

We conducted a search of articles in the PubMed, Web of Science and EBSCO Information Services using the following keywords: cancer care, physical activity, survivors, quality of life, QoL. The inclusion criteria were as follows: articles in English, publication between 2017 and 2022. A total of 971 records were initially identified. We removed 381 duplicates and excluded articles to which we had no access, articles in a language other than English and articles not directly related to the subject of the review (*n* = 326). During the eligibility assessment, we also excluded articles concerning cancer prevention, since the aim of this review was to evaluate the role of physical activity in patients already diagnosed with cancer, as well as articles with insufficient data (*n* = 228). Thirty-six articles were ultimately identified as eligible for inclusion. These publications were meta-analyses, systematic reviews and randomised controlled trials. The identification process of eligible studies is shown in Figure 1. Patient-reported outcomes were assessed using Functional Assessment of Cancer Therapy Scale (FACT) with a subscale for fatigue (FACT-F) [8]. The FACT Measurement System consists of over 250 questions, and it measures health-related QoL in patients with cancer and other chronic diseases. Patients are asked to answer about 60 questions, based on the general version FACT-G, then new questions could be added to focus specifically on the problems of a given disease [14,15,16].

## 3. Results

**Figure 2:**
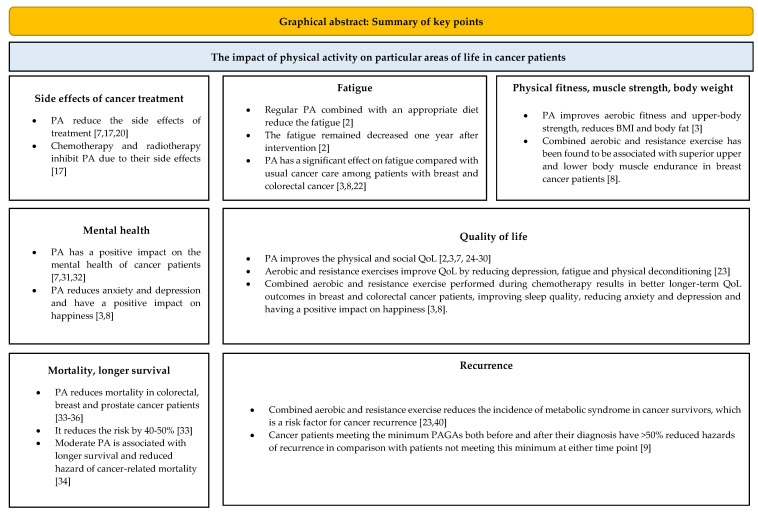
A graphical abstract summarizing the presented results.

We carried out a review of 36 systematic reviews, meta-analyses and randomised controlled trials concerning physical activity and cancer care. The studies included in the review investigated the effects of physical activity, as measured by a number of questionnaires assessing the type, frequency and duration of particular activities. We presented the results in Table 1. This table compares the influence of physical activity on cancer patients: both survivors and patients under active oncology treatment. It shows results depending on: the type of cancer, type of intervention and its intensity and the frequency and duration of PA. The main findings in Table 1 describes if the cancer care with PA is superior to the usual care of these patients. This paper presents the findings from studies investigating the impact of physical activity on particular areas of life in cancer patients, such as: QoL, mental health, physical fitness, muscle strength and impact on body weight. The survey describes the impact of PA on side effects, fatigue, mortality, survival and recurrence of cancer. A summary of the key points is presented in Figure 2.

### 3.1. Side Effects of Cancer Treatment

Chemotherapy and radiotherapy inhibit physical activity due to their side effects, such as severe fatigue, lack of energy as well as hair loss and mental health problems [17]. Chemotherapy is more likely than chemoradiotherapy to cause fatigue and reduce motivation to exercise. While chemoradiotherapy involves a more intensive treatment schedule, it is better tolerated by patients [18]. Physical activity has been shown to reduce the side effects of treatment and fatigue in cancer patients. The reduction was seen in those patients who, despite the side effects of treatment, underwent physical activity [7]. Studies report that regular PA reduces disease-specific side effects in patients with MM [19,20]. However, there is no evidence that physical activity mitigates the cardiotoxicity induced by cytostatic drugs [21].

### 3.2. Fatigue

One study included in the review found that regular physical activity combined with an appropriate diet (the patients completed 71% of the aerobic exercise sessions of 41 ± 25 min and 58% of the resistance exercise sessions planned as part of the intervention) reduced the fatigue resulting from intensive cancer treatment. The QoL was improved as well as lower limb muscle mass and endurance in breast cancer patients undergoing chemotherapy or radiotherapy. An important finding from the study was that the beneficial effect on QoL and fatigue persisted one year after the intervention [2]. Combined aerobic and resistance exercise has been found to reduce fatigue in patients with breast cancer [8]. In a study by Singh et al. [3], analysing the findings from 19 clinical trials, physical activity was observed to have a significant effect on fatigue in patients with colorectal cancer as compared with usual cancer care. Physical activity reduces the level of fatigue in cancer patients. The association between exercise and reduced fatigue has been demonstrated in patients with breast, prostate, colon and lung cancers [4]. Moreover, moderate-intensity physical activity has been found to reduce cancer-related fatigue in patients with colorectal cancer [22].

### 3.3. Quality of Life

Physical activity improves physical and social QoL and reduces anxiety and depression in cancer patients [2,3]. Unlike moderate to vigorous intensity physical activity, sedentary time negatively affects QoL and wellbeing of cancer patients [4]. Findings from one randomised controlled trial showed that aerobic and resistance exercise improves QoL by reducing depression, fatigue and physical deconditioning, which are the most common symptoms reported by breast cancer survivors [23]. Combined aerobic and resistance exercise performed during chemotherapy results in better longer-term QoL outcomes in breast and colorectal cancer patients, improving sleep quality, reducing anxiety and depression and having a positive impact on happiness [3,8]. Our review also included studies investigating the effects of physical activity on QoL in paediatric cancer patients with the use of the Paediatric Quality of Life Inventory. The studies showed that exercise interventions significantly improved QoL in the patients [24,25,26,27], even patients with haematological malignancies such as multiple myeloma [28,29,30]. Physical activity has also been shown to improve QoL and reduce anxiety and depression in ovarian cancer patients [27]. The findings from one study indicated that physical activity improves QoL in cancer patients despite the bothersome side effects of cancer treatment [7].

### 3.4. Mental Health

Physical activity has a positive impact on the mental health of cancer patients and adds positivity to their daily life [7]. One study showed that aerobic, resistance and flexibility exercises undertaken by prostate cancer patients with bone metastases for 3 months resulted in self-reported improvements in physical functioning, which had a positive influence on the mental health of the patients studied [31]. Another study found that an 8-week exercise intervention programme consisting of twice-per-week sessions of 60 min of resistance, flexibility and cardiorespiratory exercises performed by patients with different types of cancer improved the capability of the patients to express positive emotions, improved their functional capacity and had a positive influence on their mental health [32].

### 3.5. Physical Fitness, Muscle Strength, Impact on Body Weight

Studies have shown that exercise improves aerobic fitness and upper-body strength and reduces BMI and body fat in colorectal cancer patients. The results of a meta-analysis conducted by Singh et al. showed a greater effect for exercise interventions lasting over 12 weeks and interventions conducted during chemotherapy in patients with colorectal cancer [3]. Combined aerobic and resistance exercise has been found to be associated with superior upper and lower body muscle endurance in breast cancer patients [8].

### 3.6. Mortality and Longer Survival

There is an association between greater physical activity and reduced mortality in colorectal, breast and prostate cancer patients, with 40–50% risk reductions observed among individuals undertaking physical activity [33]. A study by Palesh et al. found that engaging in moderate physical activity was associated with longer survival and reduced hazard of cancer-related mortality in patients with advanced breast cancer [34]. In their study, Di Maso et al. noted that only vigorous physical activity had the advantage over inactivity in terms of reduced risks of cardiovascular and cancer mortality [35]. The cohort studies referred to by the authors reported approximately 40% reduction in mortality from prostate cancer in physically active men. Physical activity has also been found to reduce the risk of mortality in breast and colorectal cancer patients [36]. Barnard et al. [37,38] reported that intense physical activity reduces insulin resistance and insulin levels, with greater effects observed for a combination of intense physical activity and a low-fat, high-fibre diet. One study reported that breast cancer patients who met the minimum physical activity guidelines (PAGAs) had lower hazards of mortality compared with physically inactive patients (HR = 0.74, 95%, CI = 0.56 to 0.96; HR—hazard ratio; CI—confidence interval) [9]. A cohort study carried out by Wang et al. [39] that investigated the effects of recreational physical activity in patients with non-metastatic prostate cancer found that engaging in ≥17.5 MET-h/week of recreational physical activity, compared with 3.5 ≤ 8.75 MET-h/week of recreational physical activity, was associated with a 31% lower risk of prostate cancer-specific mortality (HR 0.69, CI 95%, *p* = 0.006), with no differences between the TNM stage of a tumour.

### 3.7. Recurrence

Combined aerobic and resistance exercise reduces the incidence of metabolic syndrome in cancer survivors, particularly breast cancer survivors. Metabolic syndrome is a risk factor for breast cancer recurrence [23,40]. A randomised controlled trial conducted among 100 breast cancer survivors, assigned either to exercise or usual care, showed an improvement in BMI and levels of circulating biomarkers, i.e., insulin, IGF-1, adiponectin and leptin, in the exercise group after the exercise intervention. An improvement in all metabolic syndrome variables persisted at the 3-month follow-up in the exercise group. Another study found that breast cancer patients meeting the minimum PAGAs both before and after their diagnosis had >50% reduced hazards of recurrence in comparison with patients not meeting this minimum at either time point. The study also found reduced hazards of recurrence for patients not meeting the minimum physical activity guidelines prior to diagnosis but who reported meeting the guidelines after their treatment (2-year follow-up) [9].

**Table 1:** Comparison of the influence of physical activity on cancer survivors.

Author (Ref.)	Type of Cancer, Number of Patients	Intervention Type, Intensity	Frequency, Duration	Main Findings
Singh B. et al. [3]	colorectal cancer (*n* = 670)	supervised and unsupervised aerobic and combined exercise	pre-treatment supervised: 1 session per week, unsupervised: 3–7 times per week for 4 weeks; during chemotherapy and post-treatment: from 1 to 7 sessions per week for 7 days to 6 months.	Superior to UC for: QoL, aerobic fitness, sleep, fatigue, reduced body fat, depression, upper-body strength (*p* < 0.05)
An K-Y. et al. [8]	breast cancer (*n* = 301)	CARE	25–30 min of aerobic exercise (*n* = 96), 50–60 min (*n* = 101), a combined dose of 50–60 min of aerobic and resistance exercise (*n* = 104), median of 17 weeks	The “combined” group was superior for: fatigue, upper and lower body endurance, body fat percentage—12-month follow up, CARE after chemotherapy may be optimal for longer-term health outcomes
Cannioto R.A. et al. [9]	breast cancer (*n* = 1340)	DELCaP [38]	from minimum PAGAs (the MET [41] hour equivalent of 150 min to moderate intensity RPA per week) to exceeding the minimum recommended range	1 year after diagnosis: reduction in recurrence and mortality
Parker N.H. et al. [18]	pancreatic cancer (*n* = 50)	aerobic exercise, full-body strengthening exercises	60 min/week—moderate intensity aerobic PA; 60 min/week of strengthening exercises, 7–25 weeks	Exercise recommendations for cancer survivors are important, but in order to reduce barriers to participation, further efforts are needed.
Dieli- Conwright C.M. et al. [23]	breast cancer (*n* = 100), overweight and obese survivors	combined aerobic and resistance exercise	16 weeks, 150 min of aerobic exercise with 2–3 days of resistance exercise training/week	Superior to usual care for QoL, fatigue, depression, muscular strength (*p* < 0.001). Three-month follow-up: outcomes remained improved.
Li W et al. [26]	childhood cancer (*n* = 222)	adventure-based training program: ice-breaking and teambuilding games, shuttle runs, rock climbing, high- and low-level rope courses and descending	4 training days: 2 weeks, 2, 4, and 6 months after randomisation	Significantly lower levels of CRF (*p* < 0.001), higher levels of physical activity (*p* < 0.001), QoL (*p* < 0.01)
Jones T.L. et al. [27]	ovarian cancer (20 articles with sample sizes from 10 to 7022)	aerobic	3 to 26-week intervention, from 75 min/week to 225 min/week	Higher health-related QoL, decreased levels of anxiety and depression, improvement in fatigue, physical and psychological health
Galvao D. et al. [31]	prostate cancer with bone metastases (*n* = 103)	resistance, aerobic, flexibility exercise	3 times per week, 60 min session, for 3 months	After 3 months—improved self-reported physical function, muscle strength. No changes for total body fat mass, fatigue (*p* = 0.964).
Cataldi et al. [32]	breast cancer (*n* = 3)	each session: cardiorespiratory, resistance, flexibility, postural education exercises	8-week programme, 60 min of exercise, 2 days per week	Measures of fatigue have decreased (*p* < 0.001), exercises improved physical fitness, functional capacity, capability to manage emotional life (*p* = 0.003), helped with dealing with the physiological and psychological side effects.
Di Maso et al. [35]	prostate cancer (*n* = 777)	occupational and recreational physical activity and Mediterranean diet	15-year follow-up	Intervention reduces mortality in PCa patients (due to lowering serum insulin levels, IGF and inflammation).
Wang et al. [39]	non-metastatic prostate cancer (*n* = 10,864)	recreational physical activity, e.g., walking, bicycling, aerobics, dancing, jogging, tennis	MET-h/week < 3.5, 3.5–8.75, 8.75, ≤17.5, >17.5	37% lower risk of PCSM among men with lower-risk tumours (Gleason score 2–7, T1–T2, *p* = 0.02), 31% lower risk of PCSM (>17.5 vs. 3.5 ≤ 8.75 MET-h/week)—no difference observed by tumour risk category
Dieli-Conwright C.M. et al. [40]	overweight and obese survivors of breast cancer (*n* = 100)	aerobic, resistance exercise	3 times per week for 16 weeks	Improved levels of insulin, IGF-1, leptin, adiponectin, BMI, skeletal mass index. At 3-month follow-up, all variables remained improved.
Tubiana-Mathieu N. et al. [42]	breast cancer (*n* = 138)	CPET, 6MWT- 6-min walk test	6 min	6MWT allows for the calculation of the required exercise intensity
Watson G.A. et al. [43]	Colorectal cancer (*n* = 832, *n* = 573) breast, colon cancer (multiple systematic reviews)	aerobic and resistance training	150 min of moderate intensity aerobic exercise in 3–5 sessions per week; resistance training—at least 2 days per week for 6–12 weeks	PA: reduces mortality and risk of recurrence in cancer survivors; improves QoL, allows maintaining a healthy weight, decreases fatigue.
Rogers L.Q. et al. [44]	breast cancer (*n* = 222)	BEAT Cancer—physical activity behaviour change intervention	PA recommendations from American Cancer Society, 12 supervised exercise sessions for the first 6 weeks, then unsupervised home-based exercises, >150 min/week of moderate to vigorous PA, 3- and 6-month follow-up	BEAT Cancer was superior to usual care for improvement in sleep quality (*p* < 0.01)

PA—physical activity; CARE—Combined Aerobic and Resistance Exercise; DELCaP—Diet, Exercise, Lifestyle and Cancer Prognosis Study; PAGA—Physical Activity Guidelines for Americans; RPA—recreational physical activity; MET—metabolic equivalent of task (minutes/hours); PCa—prostate cancer; PCSM—prostate-cancer-specific mortality; CRF—cancer-related fatigue.

## 4. Discussion

A diagnosis of cancer has a profound impact on the life of the patient. The fear of cancer progression, metastases and side effects of systemic treatment affects the quality of life as well as the mental and physical health of cancer patients. The anxiety, depression and bothersome somatic symptoms, such as fatigue, nausea, vomiting and hair loss, experienced by cancer patients significantly inhibit their physical activity. The barriers to undertaking physical activity faced by cancer patients are a very complex issue. They are associated with a number of factors. The nature, type and extent of cancer; the presence of metastases; cancer treatment and its side effects; the patient’s attitude to their illness and their coping strategy, as well as social and family support, have an enormous impact on the patient’s motivation and quality of life and thus their attempt to undertake regular physical activity. Moreover, cancer patients are often concerned that physical activity could have a negative impact on their illness, especially patients with diagnosed multiple myeloma, whom have the highest physical and mental impairments and a low QoL [20,45]. Furthermore, they are less willing to include exercise in their standard cancer treatment because of the fear that it will make them feel worse and due to a lack of knowledge of the benefits of physical activity. However, numerous studies have reported that standard cancer care combined with physical activity is superior to standard pharmacological care. Physical activity improves the daily functioning of cancer patients, reduces fatigue, side effects of intensive treatments, anxiety and depression and improves muscle endurance and mass, thereby allowing patients to perform their daily activities without difficulty. Moreover, the findings from the studies showed that physical activity is associated with a reduced risk of cancer of the breast, colon, stomach and endometrium (10–20% risk reduction). The studies manifest that PA reduces the risk of mortality by 40–50% for breast, colon and prostate cancers [33].

Cancer-related fatigue is a serious and complex problem that affects the quality of life and daily activities of cancer patients. Although, based on the results in the studies [2,3,4], it can be concluded that there is a correlation between fatigue and a tendency to have less PA, it cannot be considered as an unequivocal cause of decline in PA. Nevertheless, fatigue has a major impact on the functioning of cancer patients, and clinicians should aim to reduce fatigue levels. Numerous studies have shown that physical activity is associated with a significant reduction in fatigue in breast, colorectal, ovarian and prostate cancer patients and multiple myeloma patients [3,8,19,22,27,31,43]. A systematic review by Cataldi et al. found that aerobic exercise is more effective than other treatments in reducing cancer-related fatigue. Their review suggested that exercise should be performed at least 2 days per week for at least 8 weeks in order to achieve the best results and showed that the effects of low- to medium-intensity exercise did not differ between women and men [46].

According to the National Comprehensive Cancer Network (NCCN) and the American College of Sports Medicine (ACSM) (2018), physical activity improves QoL and physiological and psychological fitness in cancer patients [46].

Chemotherapy and radiotherapy have a negative impact on many aspects of the lives of cancer patients, reducing their interest in physical activity and decreasing the effectiveness of exercise. The side effects of treatment are bothersome, especially for patients with MM, and their intensity is much higher than people with other haematological cancers [20,47]. One study revealed that cancer patients found it very difficult to engage in physical activity in public places due to the side effects of their treatment, such as hair loss, as well as the fear of overheating and infection [17]. However, physical activity has been shown to reduce the side effects of cancer treatment. Importantly, the beneficial effect of an intervention involving physical activity in reducing such side effects of cancer treatment as fatigue persisted one year after the intervention [2]. Chemotherapy not only affects QoL and causes bothersome side effects, but it also has a direct impact on the patient’s physiology. It reduces mitochondrial function by impairing oxidative phosphorylation, resulting in sarcopenia. Moreover, it may reduce lung function [43,48]. It has been shown that aerobic exercise mitigates the impact of cancer treatment on physiological functions. Physical activity helps increase blood flow, activates the sympathetic nervous system, regulates the endocrine system and mobilises cytotoxic lymphocytes and NK cells, thus exerting antitumor effects. Moreover, it reduces the levels of lactate, which are a factor in promoting tumour growth [49,50].

The results from a study by Cannioto et al. [9] showed that breast cancer patients meeting the minimum guidelines for physical activity both before and after diagnosis had >50% reduced hazards of cancer recurrence and mortality. These findings are of great importance for the development of clinical oncology, as they suggest that clinicians should advise their patients to increase their physical activity immediately after a diagnosis, which would result in significant benefits. However, the benefits of regular engagement in physical activity are not only directly associated with cancer care but also translate into a reduced risk of comorbidities, improved cardiovascular function and physical fitness and thus improved wellbeing and better daily functioning.

Physical activity can improve immune system function by mobilizing leukocytes with increased functional capacities into the circulation. It helps with the elimination of dysfunctional T cells and improves the abundance of some T cell populations. PA may have an impact on CTLA-4 (inhibitory immune checkpoint) and provide to better response to immunotherapy in cancer patients [51,52].

As for incorporating exercise into cancer care and improving treatment outcomes, it is crucial to understand the role of the intensity, dose and mode of exercise in cancer patients. It is necessary to consider the individual needs of patients, the type of cancer they have as well as their treatment and health history. It has been found that the sooner physical activity is incorporated into a patient’s treatment plan after diagnosis, the more effective it is [53]. High-intensity exercise is not contraindicated for all cancer patients. Therefore, patients should not be restricted to exercise of low intensity. High-intensity exercise should be avoided by those who suffer from nausea and vomiting as well as those who have a blood clot related to a peripheral central catheter [54]. Positive effects of exercise are observed with sessions of at least 20 min on most days of the week (accounting for planned days of rest and unplanned days of inactivity [55] due to one of the following barriers: fatigue, pain, lack of motivation [54]). Recommendations from the ACSM, the NCCN and the Clinical Oncology Society of Australia (COSA) recommend participation in 150 min of moderate-intensity aerobic exercise, 3–5 sessions per week, as well as resistance training at least 2 days per week as part of a programme lasting 6–12 weeks [56,57,58]. It is also recommended that exercise interventions should, at least initially, be supervised by exercise trainers or physical therapists [59]. Cataldi et al. recommend increasing the quantity and quality of exercise in cancer patients by monitoring all parameters during exercise sessions. Persons responsible for cancer care should take into consideration the outcomes of studies on this subject, so as to best plan the intensity and volume of exercise for their patients [46]. Similarly, the World Cancer Research Fund (WCRF) and the American Institute for Cancer Research (AICR) recommend participation in at least 150 min of moderate-intensity exercise per week, including strength training exercises at least twice a week [60].

A major challenge for cancer patients is the very initiation of regular physical activity. This, in turn, is influenced by their strategy for coping with the illness. Strategies for coping with cancer can be constructive (e.g., fighting spirit, positive redefinition) or destructive (e.g., helplessness, hopelessness, anxious preoccupation). Choosing a constructive strategy will help initiate and maintain physical activity, whereas destructive strategies are a major barrier to the initiation of physical activity. The helplessness and anxiety associated with a diagnosis of cancer result in the patient giving in to the illness. This reduces the patient’s QoL, making it more difficult for them to maintain motivation for engaging in physical activity [61]. Other barriers to participation in physical activity reported by cancer patients include: fatigue, business and the associated lack of time [62], severe pain and social and environmental barriers—lack of an exercise partner, lack of exercise facilities, fear of injury, lack of willpower, lack of interest, lack of equipment and lack of experience [63,64]. A relatively large proportion of patients (approximately 17.9%) cite the lack of access to information about how to exercise and what type of exercise would be best for them as the reason for which they do not engage in physical activity [64]. Another reason why patients do not initiate physical activity is their concern that a given type of exercise is contraindicated for them due to their illness.

It is very difficult for patients to maintain the appropriate intensity of exercise, especially if they suffer from chronic comorbidities or experience bothersome side effects of cancer treatment. The occurrence of comorbidities such as hypertension, kidney disease, diabetes, liver disease or obesity is increasing in cancer survivors [65]. Obesity, which occurs particularly in colorectal and breast cancer survivors, increases the risk of heart diseases and hypercholesterolemia and has an influence on survival [66]. Attempts are being made to determine what training intensity would be most beneficial for such patients in terms of improving their QoL and maintaining their motivation for participating in physical activity. All members of the cancer care team should promote physical activity at all stages of cancer treatment. Exercise should be individualised, planned and tailored to the individual patient and adjusted to a specific type of cancer, as it offers major potential for reducing cancer morbidity and mortality [67]. Studies show that flexible time for PA sessions, low-cost and close location to home met with highest interest from patients and better compliance [20]. The literature discussed above suggests that physical activity has a significant multidimensional impact on the quality of life of cancer patients and plays a major role in improving cancer care, treatment outcomes, increasing survival time and reducing mortality in cancer patients. Therefore, it is important that clinical recommendations focus on educating patients and attempting to change their attitude to exercise [62]. It is extremely hard to find the best way to encourage patients to start and maintain physical activities. Nevertheless, the healthcare providers should aim to encourage patients to exercise. An adequate education and demonstration PA advantages may be the first step to motivate them. Healthcare professionals should devote their time to patients, list the barriers the cancer patients and cancer survivors are struggling with and should try to find a solution to reduce the barriers and recommend an appropriate intervention. Psychological help could be invaluable. The results presented in this study may be helpful to convince patients that PA can offer them many benefits for their QoL, everyday functioning and survival time.

## 5. Limitations

The study has potential limitations. The first limitation is the selection of articles only in English, which introduces a language bias. The reason for this limitation is the insufficient knowledge of other languages to discuss the results in the study with appropriate precision. The second limitation is exclusion of the papers which the authors had no access to, which may potentially have impact on the results. The third limitation is the lack of an unequivocal way to encourage patients to start and maintain PA. Our purpose is to motivate the authors of future studies to search for an effective method encouraging patients to exercise.

## 6. Conclusions

Physical activity improves quality of life, increases survival and reduces mortality, fatigue, side effects of treatment and the risk of recurrence.

Physical activity should be selected individually, depending on the type of cancer, treatment and comorbidities.

It is extremely difficult to determine what type, intensity and duration of physical activity is likely to have the greatest effect.

## Data Availability

All the data analysed during the current study are available from the corresponding author upon reasonable request.
